# A Smartphone-Based Chemosensor to Evaluate Antioxidants in Agri-Food Matrices by In Situ AuNP Formation

**DOI:** 10.3390/s21165432

**Published:** 2021-08-12

**Authors:** Donato Calabria, Massimo Guardigli, Paolo Severi, Ilaria Trozzi, Andrea Pace, Stefano Cinti, Martina Zangheri, Mara Mirasoli

**Affiliations:** 1 Department of Chemistry “Giacomo Ciamician”, Alma Mater Studiorum-University of Bologna, Via Francesco Selmi 2, I-40126 Bologna, Italy; donato.calabria2@unibo.it (D.C.); massimo.guardigli@unibo.it (M.G.); p.severi@hotmail.it (P.S.); ilaria.trozzi2@unibo.it (I.T.); andrea.pace7@unibo.it (A.P.); martina.zangheri2@unibo.it (M.Z.); 2 Interdepartmental Centre for Industrial Research in Renewable Resources, Environment, Sea and Energy (CIRI FRAME), Alma Mater Studiorum-University of Bologna, Via Sant’Alberto 163, I-48123 Ravenna, Italy; 3 Interdepartmental Centre for Industrial Aerospace Research (CIRI Aerospace), Alma Mater Studiorum-University of Bologna, Via Baldassarre Canaccini 12, I-47121 Forlì, Italy; 4 Department of Pharmacy, University Naples Federico II, Via Domenico Montesano 49, I-80131 Naples, Italy; stefano.cinti@unina.it; 5 BAT Center−Interuniversity Center for Studies on Bioinspired Agro-Environmental Technology, University of Napoli “Federico II”, I-80055 Portici, Italy; 6 Interdepartmental Centre for Industrial Agrofood Research (CIRI Agrofood), Alma Mater Studiorum-University of Bologna, Via Quinto Bucci 336, I-47521 Cesena, Italy; 7 Interdepartmental Centre for Industrial Research in Advanced Mechanical Engineering Applications and Materials Technology (CIRI MAM), Alma Mater Studiorum-University of Bologna, Viale Risorgimento 2, I-40136 Bologna, Italy

**Keywords:** total antioxidant capacity, 3-D printing, smartphone, chemosensor, gold nanoparticles, extra virgin olive oil, green tea, point-of-need, gallic acid, polyphenols

## Abstract

In recent years, there has been a continuously growing interest in antioxidants by both customers and food industry. The beneficial health effects of antioxidants led to their widespread use in fortified functional foods, as dietary supplements and as preservatives. A variety of analytical methods are available to evaluate the total antioxidant capacity (TAC) of food extracts and beverages. However, most of them are expensive, time-consuming, and require laboratory instrumentation. Therefore, simple, cheap, and fast portable sensors for point-of-need measurement of antioxidants in food samples are needed. Here, we describe a smartphone-based chemosensor for on-site assessment of TAC of aqueous matrices, relying on the antioxidant-induced formation of gold nanoparticles. The reaction takes place in ready-to-use analytical cartridges containing an hydrogel reaction medium preloaded with Au(III) and is monitored by using the smartphone’s CMOS camera. An analytical device including an LED-based lighting system was developed to ensure uniform and reproducible illumination of the analytical cartridge. The chemosensor permitted rapid TAC measurements of aqueous samples, including teas, herbal infusions, beverages, and extra virgin olive oil extracts, providing results that correlated with those of the reference methods for TAC assessment, e.g., oxygen radical absorbance capacity (ORAC).

## 1. Introduction

Antioxidants are molecules capable of slowing down, counteracting, or neutralizing the action of free radicals, thus protecting the body from oxidative stress and related diseases [1]. A variety of processes in the body (e.g., food digestion, hepatic drug metabolism, exposure to UV rays, cigarette smoke, and environmental pollutants) can produce free radicals, mostly reactive oxygen species (ROS) that include both oxygen radicals, such as superoxide anion (O_2_^•–^), hydroxyl (^•^OH), peroxyl (ROO^•^), and alkoxyl (RO^•^) radicals, as well as non-radical species that are oxidizing agents or can be easily converted into radicals, such as hypochlorous acid (HOCl), peroxynitrite (ONOO^–^), singlet oxygen (^1^O_2_), and hydrogen peroxide (H_2_O_2_). ROS are highly reactive intermediates and can therefore harm cell components, such as DNA, proteins, and lipids. When body antioxidants, either endogenous (such as superoxide dismutase and catalase enzymes, glutathione, and coenzyme Q) or introduced with the diet, are not sufficient to counterbalance the production of ROS, oxidative stress occurs. A persistent oxidative stress condition can lead to the onset of various non-communicable diseases (NCDs), such as neurodegenerative diseases (e.g., Parkinson’s and Alzheimer’s diseases, amyotrophic lateral sclerosis), atherosclerosis, and cancer [2].

Recent progress in medicine and nutrition is shifting the traditional healthcare approach towards precision medicine, which prioritizes diseases’ prevention and health promotion mainly through lifestyle and diet-based approaches [3]. In this context, plant-derived antioxidants, such as flavonoids, vitamins, hormones, phenolic acids, and esters, are considered bioactive dietary compounds able to reduce oxidative stress and have been associated with several health benefits [4]. There is therefore a growing trend in consumer preferences towards natural antioxidants and in the industrial use of antioxidant-rich plant extracts in food and beverage formulations. Furthermore, antioxidants play a key role in preserving food quality, as they slow down oxidative degradation processes during food processing, storage, and distribution [5,6].

Important groups of plant-derived antioxidants include polyphenols, carotenoids, isothiocyanates, catechins, and vitamins E and C. In green tea, where the polyphenol compounds account for 24–36% in dry weight [7], catechins are the main antioxidant components, accounting for 30–40% of total tea polyphenols [8]. In particular, (−)-epigallocatechin gallate represents up to 60–65% of the total catechins in tea [8]. Ginger (*Zingiber officinale*) root and *Aloe vera*, both rich in polyphenols (about 10–40 mg/100 g for ginger [9] and about 300 mg/100 g for aloe’s lyophilized leaf epidermis [10]), are examples of herbs that provide health benefits thanks to their high antioxidant activities [11,12]. Extra virgin olive oil (EVOO) displays many healthy properties, principally ascribed to minimal constituents in the unsaponifiable fraction, such as phenolic compounds, phytosterols, tocopherols, and pigments [13]. EVOO’s polyphenols have been shown to protect blood lipids against oxidative stress and in 2011, the European Food Safety Authority (EFSA) approved a claim on this [14]. Olive oils contain 50 to 800 mg/kg of polyphenols, the most abundant compounds being oleuropein and its breakdown derivatives (hydroxytyrosol and tyrosol), although other components also contribute to EVOO’s anti-inflammatory effects [15]. Since antioxidants can be easily degraded during food processing and storage, monitoring their activity along the food supply chain is useful for preserving food quality and health benefits [16].

A variety of methods have been developed for the evaluation of the total antioxidant capacity (TAC) of complex samples, such as food and food extracts, beverages, and biological fluids. These tests usually measure the ability of antioxidants to quench radical species through either the transfer of hydrogen atoms (HAT mechanism) or electrons (ET mechanism) from antioxidants to free radicals. Most such methods rely on spectrophotometric measurements, including the 2,2′-azino-bis(3-ethylbenzothiazoline-6-sulfonic acid (ABTS) radical scavenging assay, ferric reducing antioxidant power (FRAP) assay, cupric reducing antioxidant capacity (CUPRAC) assay, and the 2,2-diphenyl-1-picrylhydrazyl (DPPH) free radical scavenging assay. Others employ fluorescence, such as the oxygen radical absorbance capacity (ORAC) assay, or chemiluminescence (CL), e.g., the total radical trapping antioxidant parameter (TRAP) and the enhanced chemiluminescence (ECL) assays [17]. It should be noted that there are no “gold standard” assays for measuring TAC, nor a unique reference antioxidant compound, thus assay results (usually expressed in terms of equivalent content of standard antioxidants, such as Trolox, ascorbic acid, or gallic acid) can vary depending on the method employed and the antioxidant used for assay calibration.

The methods reported above are often expensive and require laboratory instrumentation, long testing time, and trained personnel. Therefore, there has been an increasing interest in the development of simple, cheap, and fast portable sensors for point-of-need measurement of TAC in food samples.

Nanomaterials have received great attention in recent years for the development of novel sensors and biosensors, owing to their unique mechanical, electrical, magnetic, optical, catalytic, and biological properties, which are size dependent and can be precisely tuned by modifying the nanoparticles’ size, shape, and extent of aggregation. Several sensors have been developed for measuring TAC of food samples employing nanomaterials as reporters (e.g., optical or electrochemical), catalysts, or immobilization platforms [18]. Gold nanoparticles (AuNPs) are particularly suited for the development of portable optical sensors, taking advantage of their stability and high extinction coefficients. In the presence of Au(III), antioxidants act as active reductants, promoting the generation and growth of AuNPs. The intensity of the AuNPs plasmon absorption bands, which account for the color of AuNPs suspensions (from red to blue depending on the particle size), correlates well with the amount of reducing species, i.e., the greater the TAC (corresponding to the tendency to donate electrons), the greater the extent of AuNPs formation. This approach has been used for the measurement of TAC in various foods, including tea, wine, fruit extracts, olive oil, chocolate, and rapeseed [19,20,21,22]. Most of these assays take place in solution and the spectroscopic measurements are made using laboratory instrumentation, thus impairing assay portability. To overcome these limitations, AuNPs-based TAC assays have also been developed in analytical formats best suited for on-site applicability, e.g., employing reagents loaded or immobilized in solid matrices. For example, Choleva et al. described a device for the determination of TAC, exploiting on-paper formation of gold nanoparticles [23]. However, despite the interesting results, this assay showed a non-homogeneous color distribution in the detection zone. In addition, image acquisition was performed using a desktop flatbed scanner instead of a portable device to assure constant lighting conditions and improve reproducibility.

To overcome these limitations, we developed a simple, inexpensive, and fast smartphone-based all-in-one chemosensor suitable for on-site TAC assessment of aqueous food matrices and extracts. In this chemosensor, the formation of AuNPs occurs in the presence of antioxidants, which act as reducing agents to promote the AuNPs nucleation and growth by a redox process [24]. The reaction gives rise to a red color proportional to the TAC of the sample, which is quantitatively assessed using gallic acid as a standard antioxidant. The chemosensor employs an analytical cartridge in which Au(III) is preloaded into a gelled agarose reaction medium; therefore, only the sample and the calibration solutions need to be added to perform the analysis. AuNPs formation is monitored using the smartphone’s built-in CMOS camera with the aid of a lighting system (a transparent optical diffuser embedding white LEDs as light sources) that ensures uniform and reproducible illumination of the analytical cartridge during image acquisition. The chemosensor permits rapid TAC measurement and provides results in good agreement with those of established methods for assessing antioxidant capacity, such as ORAC.

## 2. Materials and Methods

### 2.1. Chemicals

Agarose, Au(III) chloride trihydrate (HAuCl_4_·3H_2_O), gallic acid, luminol, sucrose, fluorescein sodium salt, 2,2′-azobis (2-amidinopropane) dihydrochloride (AAPH), and horseradish peroxidase (HRP) were purchased from Sigma Aldrich (St. Louis, MO). All other chemicals were of the highest analytical grade.

Teas, herbal infusions, and aloe antioxidant drink (containing 30% of *Aloe vera* juice and pulp) are commercially available products and were purchased from a local store. Extra virgin olive oil (EVOO) samples and EVOO waste product extracts (oleuropein-enriched extracts from olive tree leaves and hydroxytyrosol-enriched extracts from olive mill wastewaters) were obtained from Italian manufacturing companies participating in the VIOLIN (Valorization of Italian OLive products through INnovative analytical tools) research project funded by Cariplo Foundation within the “Agroalimentare e Ricerca” (AGER) program.

### 2.2. Sample Preparation

Tea and herbal infusions were prepared as follows: 1 g of solid was suspended in 20 mL of deionized water pre-warmed at 80 °C and incubated for 15 min, then the infusion was left to cool at room temperature. Subsequently, 1 mL of infusion was centrifuged at 15,000× *g* for 10 min then appropriately diluted with deionized water for the analysis.

The aloe antioxidant drink sample was centrifuged at 15,000× *g* for 10 min, then appropriately diluted with deionized water for the analysis.

EVOO samples were subjected to extraction to recover the polyphenolic fraction following a slightly modified literature procedure [25]. Briefly, 1 g of EVOO was dissolved in 1 mL of n-hexane and extracted with 4 × 1 mL of 60:40 (*v*/*v*) methanol/water. Then, 2 mL of n-hexane were added to the methanol/water extract and the mixture was vortexed and centrifuged for 5 min at 3000× *g* to remove trace lipids. Next, 2 mL of the methanol/water layer were transferred into a glass tube. Methanol was removed in a vacuum concentrator centrifuge (UNIVAPO 100H, UniEquip GmbH, Munich, Germany) under reduced pressure and the residual solution was lyophilized (Alpha 1–2 LDplus, Martin Christ GmbH, Osterode am Harz, Germany). The lyophilized extracts were resolubilized in 250 μL of DMSO, then appropriately diluted with deionized water for the analysis.

Solid EVOO by-product extracts, obtained following a published procedure [26] and kindly provided by Prof. Annalisa Romani (Laboratorio PHYTOLAB, DiSIA, University of Florence, Florence, Italy), were dissolved in deionized water and appropriately diluted for the analysis.

### 2.3. Determination of Total Antioxidant Capacity by Oxygen Radical Scavenging Capacity (ORAC) Assay

The measurement of TAC with the ORAC assay was performed according to Huang et al. [27] with slight modifications. First, 50 μL of either deionized water (blank), gallic acid standard solutions (up to 70 µM) in deionized water, or sample dilutions were transferred in triplicate in a black 96-well microtiter plate. After incubation at 37 °C for 10 min, 50 μL of fluorescein sodium salt solution (25 ng/mL in deionized water) were added to each well and the microtiter plate was incubated at 37 °C for another 20 min. Then, 25 μL of a 60 mg/mL solution of AAPH in deionized water were added to each well and the degradation of fluorescein was monitored by measuring the fluorescence signal (excitation at 485 nm and emission at 535 nm) at 5-min intervals for 1 h using a Varioskan LUX multimode microplate reader (Thermo Fisher Scientific, Waltham, MA, USA). Using the data obtained from the wells containing the gallic acid standard solutions, a calibration curve was generated by plotting the area under the fluorescence curve (upon subtraction of the area under the blank curve) against the gallic acid concentration and the experimental data were fitted with a linear equation. The TAC of the sample dilutions was then obtained by interpolating their fluorescence readings, calculated as described before, on the calibration curve. Finally, the TAC of each sample expressed in equivalents of gallic acid was estimated by averaging the values measured for each dilution upon correction for the respective dilution factor. Data were presented as mean ± SD of three experiments.

### 2.4. Determination of Total Antioxidant Capacity by the Enhanced ChemiLuminescence Method

The ECL method for TAC measurement is based on the competition between the reaction of peroxyl radicals with luminol, giving rise to light emission, and the scavenging of radicals by antioxidants. Indeed, the addition of antioxidants to a glowing steady-state luminol CL reaction temporarily quenches light output. The duration of light emission quenching is related to the amount and the strength of the antioxidant added [28]. Assay was performed in black 96-well microtiter plates. A CL cocktail was prepared by adding 100 µL of a 1:100,000 (*v*/*v*) dilution of a stock HRP solution (1 mg/mL in 0.1 mol/L Tris-HCl buffer, pH 7.4) to 10 mL of a 1:1 (*v*/*v*) mixture of Luminol/Enhancer and Peroxide Chemiluminescent Detection Reagent solutions (ECL Western Blotting Reagent, Merck KGaA, Darmstadt, Germany). Then, 10 μL of gallic acid standard solutions (up to 70 μM) for the generation of the calibration curve, sample dilutions, or water (as negative control) were dispensed in triplicate in the wells of the microtiter plate, then 100 μL of the CL cocktail were added to each well. The kinetics of light emission was monitored at time intervals of 20 s for 60 min using a Varioskan LUX multimode microplate reader. For each well (except the control), the emission inhibition time, defined as the time in which the CL signal reaches a value equal to half of the maximum signal in that well, was determined by analyzing the CL kinetic curves. Using the data obtained from the wells containing the gallic acid standard solutions, a calibration curve was constructed by plotting the duration of emission inhibition versus the concentration of gallic acid, and then the TAC of sample dilutions was calculated by interpolating their inhibition times on the calibration curve. Finally, the TAC of each sample expressed in equivalents of gallic acid was obtained by averaging the values measured for each dilution upon correction for the respective dilution factor. Data were presented as mean ± SD of three experiments.

### 2.5. Antioxidant Activity Chemosensor

The TAC chemosensor consists of two components, the disposable analytical cartridge containing all the reagents required to perform the assay and the analytical device, which provides illumination and optics for imaging the analytical cartridge with the smartphone during the assay.

#### 2.5.1. Antioxidant Activity Chemosensor

**Figure 1:**
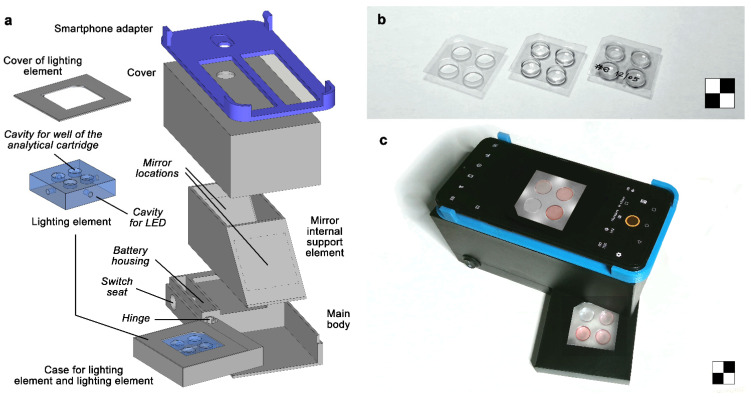
(**a**) Schematic drawing of the 3-D-printed elements of the analytical device (electric components, such as battery, LED control switch, LEDs, and electrical wiring, are not shown); (**b**) From left to right: vacuum-thermoformed analytical cartridge, analytical cartridge filled with the gelled reaction medium, analytical cartridge sealed with the adhesive plastic foil for storage; (**c**) Analytical device connected to the OnePlus 6 smartphone. The checkerboards are 2 × 2 cm.

The analytical cartridge (Figure 1b) consists of a vacuum-thermoformed PVC sheet (35 × 35 mm, thickness 0.18 mm) with four round wells (diameter 10 mm, depth 4.0 mm). A corner of the cartridge is cut to constrain the orientation of the cartridge when inserted in the analytical device. Each well is filled with 150 µL of an agarose-based gelled reaction medium containing 6 mg/mL of agarose, 0.30 mg/mL of Au(III) chloride, and 10 mg/mL of sucrose as AuNPs stabilizer. To prepare the reaction medium, agarose was first dissolved in hot (80 °C) deionized water, then sucrose and Au(III) chloride were added and the mixture was stirred until complete dissolution. The hot solution was then dispensed into the wells of the analytical cartridge and left to cool to room temperature to obtain the gelled reaction medium. Finally, the cartridges were sealed with an adhesive plastic foil to avoid the dehydration of agarose gel and stored in the dark at 4 °C until use.

#### 2.5.2. Assay Device

The 3-D-printed components of the assay device (Figure 1a) were designed using the SketchUp Pro 2021 CAD software (Trimble Inc., Sunnyvale, CA, USA). The device has an overall size of 140(W) × 70(H) × 70(D) mm (not including the smartphone adapter, the size of which may vary according to the smartphone model) and was produced by stereolithography (SLA) using a Form 2 3-D stereolitography (SLA) desktop printer (Formlabs Inc., Somerville, MA, USA). The 3-D-printed components were mostly manufactured in black photopolymerizable resin to avoid undesired light reflections and include: (a) a main body, which houses an A27 12V alkaline battery and a switch for powering the LEDs of the lighting element; (b) a case for the lighting element, connected to the central body with a hinge for easy insertion of the analytical cartridge, and a cover to shield the direct light emission of the LEDs; (c) an internal mirror support element with two 40 × 40 mm flat mirrors angled at 45°; (d) a cover to avoid interference from ambient light during the measurement and to support the smartphone adapter; and (e) a smartphone adapter designed for the OnePlus 6 smartphone (OnePlus, Shenzen, China). The lighting element is manufactured in clear photopolymerizable resin and sanded to improve the light scattering capacity. It has four upper cavities to accommodate the wells of the analytical cartridge. Four further small cavities in the lateral faces of the lighting element house the cool white LEDs (color temperature 4000–4500K) that provide illumination of the cartridge. Figure 1c shows the analytical device connected to the OnePlus 6 smartphone in use.

### 2.6. Analytical Procedure for the Quantification of Antioxidant Activity

The analytical cartridge was allowed to warm to room temperature, then the covering adhesive plastic foil was removed. Next, 100 µL of blank (deionized water), of the gallic acid standard solutions (250 and 500 µM gallic acid in deionized water), or of sample (properly diluted in deionized water) were pipetted into the wells of the cartridge. After 20 min of incubation at room temperature, the cartridge was inserted into the device, the LEDs were turned on, and the image of the cartridge was acquired with a dedicated app (Camera FV-5 Lite, freely available from Google Play) using ISO 800 sensitivity and 0.1 s exposure time and saved in TIFF format. The image was then analyzed using the freeware software ImageJ v.1.53h (National Institutes of Health, Bethesda, MD, USA). Regions of interest (ROIs) corresponding to the inner part of the wells of the analytical cartridge were defined and for each ROI, the average RGB values were calculated. Then, the saturation value (S) in the HSV color space was obtained for each well by the formula:

(1) $$S=\frac{\max\left(\mathrm{RGB}\right)-\min\left(\mathrm{RGB}\right)}{\max\left(\mathrm{RGB}\right)}$$

A three-point calibration curve was generated by plotting the S values measured for the blank and the two gallic acid standards against the gallic acid concentration and the experimental data were fitted with a linear function. Finally, the TAC of the sample expressed in equivalents of gallic acid was calculated by interpolating its S value on the calibration curve. Data were presented as mean ± SD of three experiments.

### 2.7. Data Analysis

All data analysis and statistical data elaboration were performed using GraphPad Prism, version 8.0 (GraphPad Software, Inc., La Jolla, CA, USA).

## 3. Results and Discussion

### 3.1. Device Design

One of the main problems in the development of colorimetric smartphone-based chemosensors is the difficulty of ensuring homogeneous illumination of the detection area [29,30]. The approaches used to date have generally been based on the use of the smartphone’s built-in flash or even of lighting LEDs, often in combination with suitable light diffusers [31]. However, it is difficult to ensure homogeneous lighting, especially when using the smartphone built-in flash (which is off-center with respect to the smartphone’s camera), and to avoid unwanted light reflections. In addition, for a widespread application of smartphone-based sensors, the analytical device should be adaptable to different smartphone models, each of them with its own camera and flash position and characteristics [32].

To overcome these limitations, we designed a device that can be very easily adapted to any smartphone and provides analytical cartridge illumination without relying on the smartphone’s built-in flash. Indeed, the device, produced by 3-D printing, is equipped with its own lighting element, designed to ensure optimal illumination conditions (Figure 1a). This part is made of transparent, colorless photopolymerizable resin with a refractive index of about 1.55 [33]. It has four upper cavities to house the wells of the analytical cartridge and is equipped with four white LEDs inserted in the side faces to illuminate the analytical cartridge from the side and from below. Furthermore, all the surfaces of the lighting element were sanded to scatter and homogeneously distribute the light produced by the LEDs.

Another critical issue to address in the development of smartphone-based chemosensors is the distance between the smartphone’s built-in camera and the detection zone. Modern smartphones can focus on objects from 4–5 cm away, or even less if equipped with macro cameras. Additional lenses can also be used to further reduce the minimum focusing distance, thus providing a compact device [30]. However, these approaches can only be used when the detection zone is relatively small. Large detection areas cannot be fully imaged, or, at best, the outermost parts of the image exhibit a high perspective distortion, which could affect the results of the measurement. This problem can be avoided by increasing the distance between the smartphone and the detection zone. This last approach was used in our device, in which two flat mirrors angled at 45 degrees increase the focal distance from 50 to more than 130 mm without compromising device compactness. This allowed imaging of the whole analytical cartridge without any significant image distortion.

As the smartphone is concerned, this chemosensor does not necessarily require a high-end mobile device. In fact, neither high image resolution nor high sensitivity are required for the measurement; therefore, even cheap smartphones can be used (we used a OnePlus 6 smartphone, which is a mid-range smartphone marketed in 2018). To increase the versatility of the device, the adapter for the smartphone, which holds the smartphone’s camera aligned with the mirrors of the device, is an independent device component. It can thus be redesigned and replaced to use any other smartphone model. Even regarding the smartphone’s built-in camera app, almost any camera management software could be used, including those provided with the smartphone. The only precaution to take is to disable any automatic image enhancement function, which could alter the color of the image.

### 3.2. Hydrogel-Based Reaction Medium

In this work, we conducted the AuNP formation reaction into a hydrogel, in order to conveniently preload Au(III) chloride in the analytical cartridge and to improve the analytical performance. Indeed, the hydrogel matrix increases AuNPs colloid stability [34], thus providing a uniform and reproducible color for the smartphone-based readout. The critical prerequisites of our vehicle for Au(III) chloride storage and AuNPs formation were environmental friendliness; inertness towards the analyte, the reagents, and the nanomaterial; and suitability as a reaction medium for AuNP formation.

Considering these requirements, agarose was chosen as the best candidate, presenting several positive features, such as low-cost, food-grade characteristics, thermo-reversible gelation, and high transparency in the visible range [35,36]. Agarose showed the ability to let the sample components permeate the gel and react with Au(III) chloride, as well as to trap the formed AuNPs, avoiding their aggregation. Moreover, its optical transparency enabled homogeneous light transmission for uniform illumination, ensuring accuracy and reproducibility in the colorimetric measurement.

### 3.3. Optimization of the Concentration of Au(III) in the Hydrogel Reaction Medium

The sensor is based on the production of AuNPs that occurs within the hydrogel in the presence of antioxidant compounds. Indeed, when added to the well, the sample solution is rapidly absorbed in the gelled matrix, where the antioxidant molecules reduce Au(III) ions to Au atoms, to form nanoclusters drawing AuNPs formation. The reduction of the metal ions is rapid and results in a homogeneous AuNP dispersion in the reaction medium, also taking advantage of the stabilizing effect of sucrose molecules, acting as a capping agent. 

**Figure 2:**
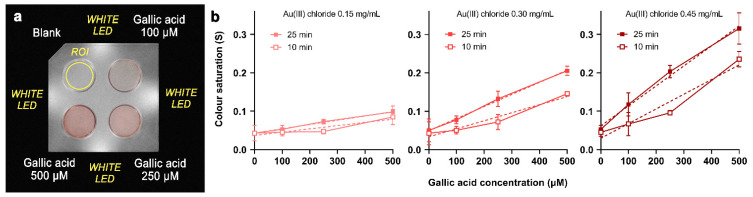
(**a**) Image of the analytical cartridge taken during the analysis; (**b**) Calibration curves obtained after 10 and 25 min of incubation in analytical cartridges prepared using 0.15, 0.30, and 0.45 mg/mL of Au(III) chloride. The dashed lines represent the linear regression fitting of the experimental data. Each point is the mean ± SD of three experiments.

As expected, the intensity of the red color due to AuNPs increases with the concentration of gallic acid (Figure 2a). The optimal concentration of Au(III) chloride acid in the agarose gelled reaction medium was determined by comparing the slope of calibration curves obtained by plotting the S values versus the concentration of gallic acid in the presence of different amounts of Au(III). As shown in Figure 2b, the color saturation value increases with the concentration of Au(III) in the hydrogel reaction medium. However, for the highest Au(III) chloride concentration tested (0.45 mg/mL), a non-homogenous color distribution within the wells was often observed, especially for the highest gallic acid concentrations. This behavior might be due to the diffusion of Au(III) ions outside the hydrogel, which, driven by the high concentration gradient, might occur before the sample volume is completely absorbed within the gel. In these conditions, a fraction of AuNPs is formed outside the solid support, thus causing inhomogeneity of the color distribution as well as possible loss of nanoparticles during cartridge handling. The incomplete retainment of reagents into the gelled agarose matrix partially compromises the assay portability and hinders an accurate evaluation of the color saturation (this is also suggested by the higher dispersion of the result shown in Figure 2b for 0.45 mg/mL Au(III) and 500 µM gallic acid). Therefore, the 0.30 mg/mL Au(III) chloride concentration was selected as the best compromise between a high assay sensitivity and uniform distribution of the produced AuNPs in the gelled reaction medium, which facilitates the evaluation of color intensity.

### 3.4. Effect of Incubation Time 

The calibration curves (Figure 2b) indicate that the color intensity, thus the slope of the calibration curve, increases with time. The production of AuNPs is faster for high gallic acid concentrations, so that at short times (i.e., at 10 min of incubation), the calibration curves are quite far from linearity. However, for longer incubation times, as the color saturation increased, the linearity of the calibration curves also improved. After 25 min of incubation, there is a good linear correlation between the color saturation and amount of gallic acid.

**Figure 3:**
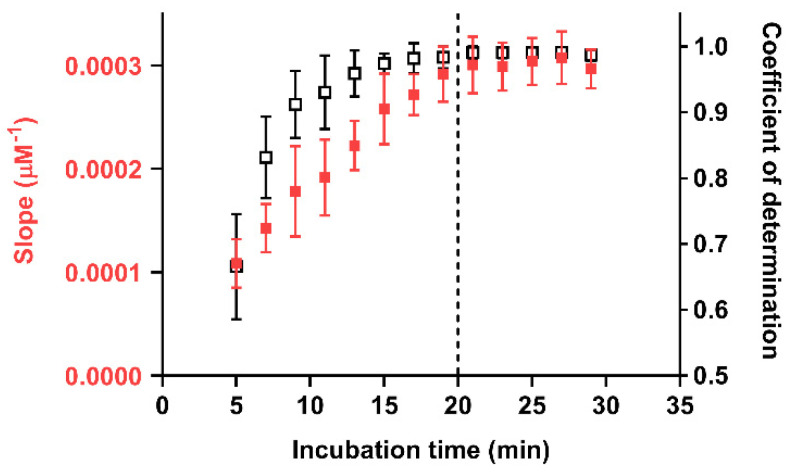
Slope (red) and linear regression coefficient of determination (black) values for calibration curves obtained at different incubation times in analytical cartridges prepared with 0.30 mg/mL of Au(III) chloride. The dashed line indicates the incubation time (20 min) selected for the assay. Each point is the mean ± SD of three experiments.

The effect of the incubation time on the quality of the calibration curve was thus studied in detail. Figure 3 shows the changes in the slope and linearity (in terms of the coefficient of determination of the linear regression) of the calibration curves obtained in analytical cartridges prepared with 0.30 mg/mL of Au(III) chloride in the time interval 5–30 min. The slope of the calibration curve increases with time up to approximately 20 min, then it remains nearly constant, indicating that AuNPs production has ceased. At the same time, the coefficient of determination R^2^ approaches unity, and for incubation times longer than 20 min, the R^2^ values are always greater than 0.98. In accordance with these results, the optimal incubation time for performing the assay was established as 20 min, since no further improvement was observed at longer times.

**Figure 4:**
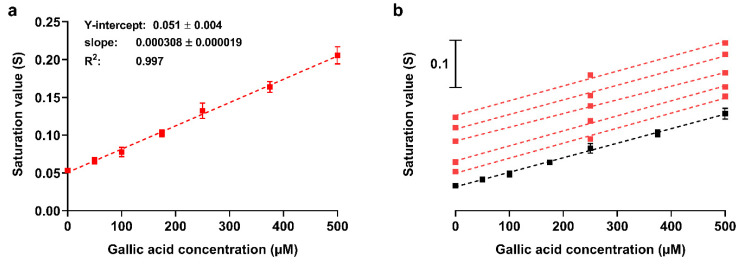
(**a**) Assay reference calibration curve obtained by analyzing gallic acid standard solutions. Each point is the mean ± SD of three experiments; the signals were not subtracted of the blank S value. (**b**) Comparison between the reference calibration curve (black) and three-point calibration curves (red) obtained in single cartridges (the curves were shifted along the Y axis to avoid overlapping). The dashed lines represent the linear regression fitting of the experimental data. Linear regression parameters for the three-point calibration curves vary from 0.046 to 0.058 (Y-intercept) and from 0.000293 to 0.000317 (slope), while R^2^ is always higher than 0.99.

To evaluate the assay performance, a reference calibration curve was generated by analyzing gallic acid standard solutions (since each cartridge allows measurement of four samples, the calibration curve was obtained using several cartridges). The calibration curve (Figure 4a) confirmed the good linear correlation between the color saturation and concentration of gallic acid in the investigated range (up to 500 µM). It also allowed estimation of the limit of detection (LOD) of the assay for TAC expressed as gallic acid equivalents, which is defined as the gallic acid concentration giving a color saturation corresponding to that of the blank plus three times its standard deviation. The LOD value (30 µM) was in the same order of magnitude of that obtained for other paper-based assays [37] and similar to that calculated from our gallic acid calibration curves for ORAC (5 µM) and ECL (30 µM). This proved that the assay is suitable to measure the TAC of teas, herbal infusions, beverages, and food extracts. As concerns sample dilution, dilution factors ranging from 1:100 to 1:1000 (*v*/*v*) depending on the TAC value proved adequate for our samples. 

Since the number of standards that can be assayed in a cartridge is limited, we assessed the reliability of calibration curves obtained using only two gallic acid standard solutions and a blank. Figure 4b shows the comparison between the reference calibration curve and a set of three-point calibration curves obtained in single cartridges using the readout of blank and two gallic acid standard solutions (i.e., 250 and 500 µM). As demonstrated by the linear regression parameters, the three-point calibration curves are reproducible and superimposable to the reference one, suggesting that the response of the chemosensor can be reliably evaluated by measurements performed in single cartridges. Thanks to the reproducibility of calibration curves, preliminary sample dilution evaluation could be performed by assaying three different sample dilutions and a blank in a cartridge. Successively, an accurate quantitative assessment will be performed by analyzing the selected sample dilution in parallel with the three-point calibration curve.

### 3.5. Measurement of Total Antioxidant Capacity of Real Samples 

The smartphone-based AuNPs assay was used to measure the TAC of aqueous real samples, including green tea, “ginger and lemon” and “ginger and orange” herbal infusions, an aloe-based antioxidant drink, EVOO extracts, and EVOO waste product extracts (either from olive tree leaves or from olive mill wastewaters). Apart from EVOO samples, which required liquid-liquid extraction of the phenolic fraction due to the fat-rich nature of the matrix, all other samples could be directly analyzed upon simple dilution and, if necessary, filtration or centrifugation to eliminate suspended solids. 

**Figure 5:**
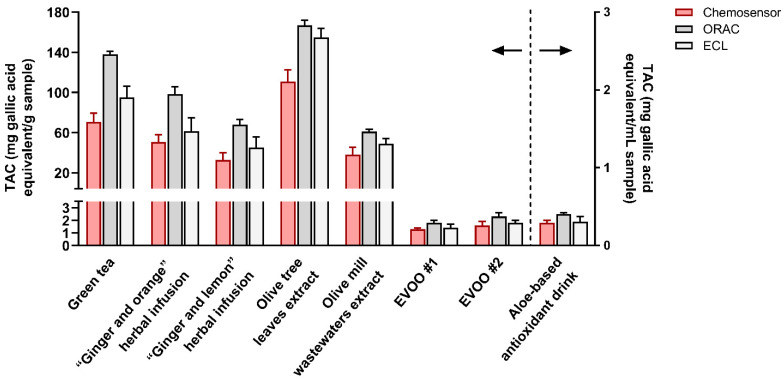
Results of TAC measurements performed in real samples with the AuNPs sensor and the ORAC and ECL antioxidant activity assays. Each data is the mean ± SD of three experiments.

The results obtained, expressed in mg gallic acid equivalent/g (or mL) of the original sample, are summarized in Figure 5.

**Figure 6:**
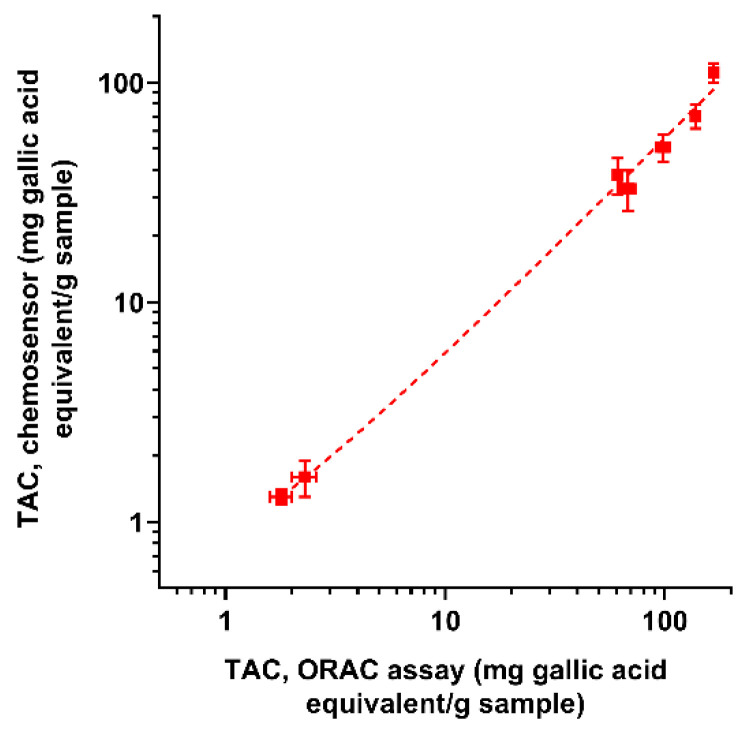
Correlation between the TAC values measured for real samples with the chemosensor and the ORAC reference assay. Each value is the mean ± SD of three experiments. Parameters of the linear regression are slope = 0.56, Y-intercept = 0.31, R^2^ = 0.98.

To assess the assay accuracy, the TAC values measured by the smartphone-based AuNPs chemosensor were compared with those obtained using the ORAC and ECL reference TAC assays (results also shown in Figure 5, while Figure 6 shows the correlation between the chemosensor and ORAC TAC results). In general, different TAC values were obtained for each sample with the three methods. This is expected, as methods are based on different reaction mechanisms [17]. In particular, while ORAC and ECL assays rely on the radical scavenging activity of antioxidant compounds in the sample, the developed sensor relies on the reducing ability of antioxidants to Au(III). The TAC values obtained with the chemosensor were appreciably lower than those of the ORAC assay but closer to those of the ECL assay. Nevertheless, for all the assays, the TAC values of the different samples showed similar trends (Figure 6), indicating that the chemosensor could reliably discriminate between samples with different antioxidant contents as well as reveal changes in TAC due to food processing or storage.

A statistical evaluation was carried out by one-way analysis of variance (ANOVA) to compare the ability of each method to discriminate between different samples. The results, as reported in Figure S1, show that our chemosensor has a discrimination ability between samples comparable to that obtained with the reference methods, and it could therefore be considered a complementary technique for TAC determination.

### 3.6. Chemosensor Stability

The chemosensor stability was evaluated, by monitoring the changes of the response to gallic acid standards using sealed cartridges stored in the dark at +4 °C for different times. The stability of the chemosensor was evaluated by measuring the changes of the analytical sensitivity (defined as the slope of the calibration curve for gallic acid generated during the analysis) during storage. The response of the chemosensor was unchanged (i.e., slope at least 90% of the initial value) for at least 14 days (Figure S2), showing the ability of the hydrogel medium to maintain the reagents’ performance. In this context, the choice of the standard antioxidant used for the generation of the calibration curve during the assay must also be considered. To facilitate on-site application of the chemosensor, gallic acid was preferred as the standard antioxidant, thanks to the easier preparation of solutions and the higher stability [38] with respect to other standards for TAC evaluation, such as ascorbic acid or Trolox.

## 4. Conclusions

In conclusion, we developed a portable device for on-site measurement of TAC of aqueous matrices, exploiting the formation of AuNPs driven by antioxidants in the presence of Au(III). The reaction was conducted in a ready-to-use cartridge, in which all the reagents are stored in a hydrogel, which also has the task of ensuring a homogeneous distribution of AuNPs and optimal color measurement. A 3-D-printed cartridge was produced to enable smartphone camera-based detection. The assay is easily and rapidly performed in a non-laboratory setting and provided results that correlate with standard TAC assays on a variety of food matrices. The developed chemosensor is suitable for monitoring the quality of food matrices along the supply chain.

## Data Availability

Data sharing not applicable. Additional data are available in the Supplementary material section.

## Supplementary Materials

The following are available online at https://www.mdpi.com/article/10.3390/s21165432/s1, Figure S1: Statistical evaluation of the between sample discrimination ability of the chemosensor, as compared with ORAC and ECL reference methods, Figure S2: Evaluation of the chemosensor long term stability.
