# Supplementary material for: A Smartphone-Based Chemosensor to Evaluate Antioxidants in Agri-Food Matrices by In Situ AuNP Formation
Source: Sensors (Basel). 2021 Aug 12;21(16):5432. doi: 10.3390/s21165432 (PMC8401892; doi:10.3390/s21165432)
Supplement: Supplementary file 1 [file sensors-21-05432-s001.zip › sensors-1321893-supplementary.pdf]

Supplementary Material to:

A smartphone-based chemosensor to evaluate antioxidants in agri-food matrices by in situ AuNP formation

Donato Calabria [1], Massimo Guardigli [1,2,3], Paolo Severi [1], Ilaria Trozzi [1], Andrea Pace [1], Stefano Cinti [4,5], Martina Zangheri [1,6,7], and Mara Mirasoli [1,2,3,]*

1 *Department of Chemistry "Giacomo Ciamician", Alma Mater Studiorum – University of Bologna, Via Francesco Selmi 2, I-40126 Bologna, Italy*

2 *Interdepartmental Centre for Industrial Research in Renewable Resources, Environment, Sea and Energy (CIRI FRAME), Alma Mater Studiorum - University of Bologna, Via Sant'Alberto 163, I-48123 Ravenna, Italy*

3 *Interdepartmental Centre for Industrial Aerospace Research (CIRI Aerospace), Alma Mater Studiorum - University of Bologna, Via Baldassarre Canaccini 12, I-47121 Forlì, Italy*

4 *Department of Pharmacy, University Naples Federico II, Via Domenico Montesano 49, I-80131 Naples, Italy*

5 *BAT Center–Interuniversity Center for Studies on Bioinspired Agro-Environmental Technology, University of Napoli "Federico II", I-80055 Portici, Italy*

6 *Interdepartmental Centre for Industrial Agrofood Research (CIRI Agrofood), Alma Mater Studiorum - University of Bologna, Via Quinto Bucci 336, I-47521 Cesena, Italy*

7 *Interdepartmental Centre for Industrial Research in Advanced Mechanical Engineering Applications and Materials Technology (CIRI MAM), Alma Mater Studiorum - University of Bologna, Viale Risorgimento 2, I-40136 Bologna, Italy*

* *Correspondence: mara.mirasoli@unibo.it*

1. Statistical evaluation of the between sample discrimination ability of the chemosensor, as compared with ORAC and ECL reference methods

A statistical evaluation was carried out by one-way analysis of variance (ANOVA) to compare the ability of each method to discriminate between different samples, i.e., to evidence a statistically significant difference between samples. The results obtained for each test on every single sample

are expressed as mean ± SD of at least three independent experiments. For each assay, differences between the means obtained on each sample were determined by one-way ANOVA followed by post-hoc Tukey multiple comparison test using GraphPad Prism, version 8.0 (GraphPad Software, Inc., La Jolla, CA). The adjusted P values were graphically reported into heat maps (Figure S1), evidencing that our chemosensor shows comparable discrimination ability with respect to the reference methods and it could therefore be considered a complementary technique for TAC determination.

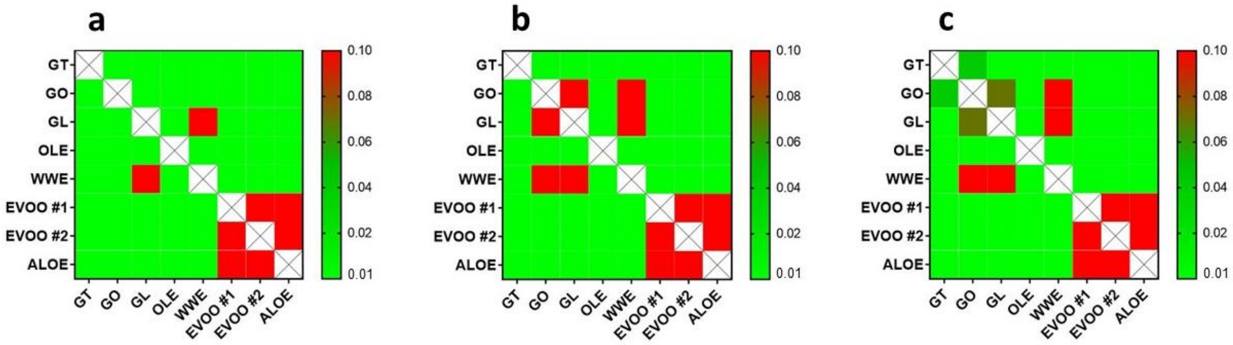

Figure S1. Heat map representing the p values obtained when performing one-way ANOVA on the mean values obtained with the tested samples employing a given analytical method (ORAC in panel a, ECL in panel b and the developed chemosensor in panel c). Samples are indicated as follows: green tea (GT), "ginger and orange" herbal infusion (GO), "ginger and lemon" herbal infusion (GL), olive tree leaves extract (OLE), olive mill wastewaters extract (WWE), extravirgin olive oil sample 1 (EVOO #1), extravirgin olive oil sample 2 (EVOO #2), aloe-based antioxidant drink (ALOE).

2. Evaluation of the chemosensor long term stability

To investigate the long-term stability of the chemosensor, we measured the changes of the analytical sensitivity (defined as the slope of the calibration curve generated during the analysis) during storage of sealed cartridges. For the stability study, a series sealed cartridges were stored in the dark at +4 °C and at regular time intervals, they were used to generate calibration curves for gallic acid. The slopes of the curves (reported in Figure S2) show that the response remains constant for at least 14 days, thus confirming the possibility to produce in advance cartridges ready for the analysis and to store them until use without any significant decrease in sensitivity.

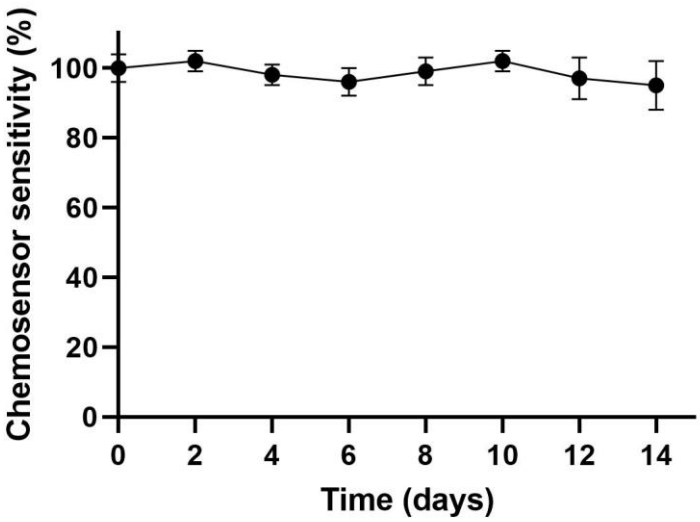

Figure S2. Changes in the sensitivity of the chemosensor upon storage of the sealed cartridges in the dark at 4°C (the sensitivity obtained at time 0 was taken as a reference). Measurements were performed in triplicate.
